# Association between active cooling and lower mortality among patients with heat stroke and heat exhaustion

**DOI:** 10.1371/journal.pone.0259441

**Published:** 2021-11-17

**Authors:** Jun Kanda, Shinji Nakahara, Shunsuke Nakamura, Yasufumi Miyake, Keiki Shimizu, Shoji Yokobori, Arino Yaguchi, Tetsuya Sakamoto

**Affiliations:** 1 Japanese Association for Acute Medicine Heatstroke and Hypothermia Surveillance Committee, Tokyo, Japan; 2 Department of Emergency Medicine, Teikyo University School of Medicine, Tokyo, Japan; 3 Graduate School of Health Innovation, Kanagawa University of Human Services, Kanagawa, Japan; 4 Emergency Medical Center, Yokohama Rosai Hospital, Kanagawa, Japan; 5 Emergency and Critical Care Center, Tokyo Metropolitan Tama Medical Center, Tokyo, Japan; 6 Department of Emergency and Critical Care Medicine, Nippon Medical School, Tokyo, Japan; 7 Department of Critical Care and Emergency Medicine, Tokyo Women’s Medical University, Tokyo, Japan; Universidade Federal de Minas Gerais, BRAZIL

## Abstract

Body cooling is recommended for patients with heat stroke and heat exhaustion. However, differences in the outcomes of patients who do or do not receive active cooling therapy have not been determined. The best available evidence supporting active cooling is based on a case series without comparison groups; thus, the effectiveness of this method in improving patient prognoses cannot be appropriately quantified. Therefore, we compared the outcomes of heat stroke patients receiving active cooling with those of patients receiving rehydration-only therapy. This prospective observational multicenter registry-based study of heat stroke and heat exhaustion patients was conducted in Japan from 2010 to 2019. The patients were stratified into the “severe” group or the “mild-to-moderate” group, per clinical findings on admission. After conducting multivariate logistic regression analyses, we compared the prognoses between patients who received “active cooling + rehydration” and patients who received “rehydration only,” with in-hospital death as the endpoint. Sex, age, onset situation (i.e., exertional or non-exertional), core body temperature, liver damage, renal dysfunction, and disseminated intravascular coagulation were considered potential covariates. Among those who received active cooling and rehydration-only therapy, the in-hospital mortality rates were 21.5% and 35.5%, respectively, for severe patients (n = 231) and 3.9% and 5.7%, respectively, for mild-to-moderate patients (n = 578). Rehydration-only therapy was associated with a higher in-hospital mortality in patients with severe heat illness (adjusted odds ratio [aOR], 3.29; 95% confidence interval [CI], 1.21–8.90), whereas the cooling methods were not associated with lower in-hospital mortality in patients with mild-to-moderate heat illness (aOR, 2.22; 95% CI, 0.92–5.84). Active cooling was associated with lower in-hospital mortality only in the severe group. Our results indicated that active cooling should be recommended as an adjunct to rehydration-only therapy for patients with severe heat illness.

## Introduction

Heat illnesses, caused by exposure to or exertion in hot environments, are a growing public health concern owing to climate change. Heat stroke and heat exhaustion are a severe form and a mild-to-moderate form of heat illnesses, respectively. In July 1995, Chicago, IL (USA) sustained a heat wave that resulted in more than 600 excess deaths and 3300 excess emergency department visits [1]. In France, more than 14 800 people died of a heat stroke caused by a heatwave in August 2003 [2]. Since 2010, an increasing number of heat stroke patients have been reported in various parts of the world [3–7].

In Japan, because of high temperatures and humidity in the summer, heat stroke and heat exhaustion occur more frequently among elderly people. More than 50,000 patients with heat illnesses are taken to hospitals each summer. On account of heat illnesses caused by the heat wave of 2018, 95,137 patients required hospital visits and 1677 patients died [8, 9]; most of these patients were older adults who were affected during daily activities [10].

Heat stroke is a life-threatening condition requiring prompt recognition and vigorous treatment [11]. Gaudio et al. [12] recommend active cooling to quickly reduce body temperature and fluid replacement to address dehydration. The best available evidence supporting active cooling is shown in a case series without comparison groups; thus, this case series alone could not appropriately quantify the effectiveness of this method in improving patient prognoses [1]. Various studies have reported improved symptoms and fewer patient fatalities after administering the following active cooling methods: cold-water immersion [13–17]; placing numerous ice-filled rubber bottles over the body [18] or covering the body with water-soaked fine gauze sheets [19]; use of fans [19], body-cooling units [20], water sprays [19, 21], and external cooling and cold-water gastric lavage [22]. The Japanese guidelines for heat stroke and heat exhaustion, created by the Japanese Association for Acute Medicine, recommend two active cooling methods in addition to evaporative methods: external convective cooling and internal gastric lavage and bladder irrigation [23]. The guidelines do not recommend using cold-water immersion, body-cooling units, intravascular ice cradles, and temperature management by extracorporeal membrane oxygenation because of safety concerns regarding older patients and those with disturbed consciousness or shock status.

Several controlled trials have shown that among healthy volunteers with exercise-induced hyperthermia, evaporative plus convective cooling or immersion in water baths at 2°C can reduce body temperature faster than other cooling methods [24, 25]. Comparisons to determine the effectiveness of various cooling methods among patients with heat illnesses are lacking.

In this study, we compared the prognosis of heat stroke and heat exhaustion patients treated with active cooling methods or with rehydration-only therapy by using data from a nationwide heat stroke and heat exhaustion registry database. We determined the therapeutic value of active cooling as an adjunct to rehydration-only therapy for the management of severe heat-related illness.

## Materials and methods

### Study settings

Japan has a temperate humid climate with four seasons. During the summer season from June to September, daytime maximum temperatures and relative humidity exceed 35°C (308.15 K) and 75%, respectively. The so-called unpleasant days, defined as a discomfort index ≥80, account for nearly 30 days in Tokyo [8]. In addition, Japan has the world’s most aged society: individuals aged ≥65 years account for 28.6% of the population, as of 2020 [26]. Most heat stroke and heat exhaustion patients are therefore older adults. Greater frequencies of heat stroke and heat exhaustion cases are seen every year. The Japanese Association for Acute Medicine started the Heat stroke Study (HsS) in 2006 as a biennial nationwide multicenter registry of heat stroke and heat exhaustion patients. The data has been collected annually since 2017. Participating facilities are emergency centers capable of providing comprehensive emergency care 24 hours a day, 7 days a week, for all serious and multidisciplinary emergencies. In Japan, there are guidelines for treating heat stroke. Each facility selects a cooling method based on its available medical equipment, supplies, and human resources.

### Study design

This study was a prospective observational multicenter registry-based study of hospitalized patients with heat stroke and heat exhaustion. We evaluated the prognoses of patients who received active cooling in hospital and patients who did not. The Ethical Review Board for Medical and Health Research at Teikyo University (Tokyo, Japan) approved the study protocol (approval numbers. 17-021-5 and 17-053-3). The study protocol was also approved by the ethical review boards of each participating institution. The requirement for informed consent was waived because of the retrospective study design and the use of anonymized data.

### Data source

We obtained the following data at hospital admission from the HsS registry: age, sex, year of registration, core body temperature, consciousness, liver damage, renal dysfunction, and disseminated intravascular coagulation (DIC). We focused on the associations between cooling methods and patients’ outcomes; therefore, we excluded the HsS data of 2006 and 2008 because they did not include this information.

The HsS data collection was conducted from July to September in the study years (i.e., 2010, 2012, 2014, and 2017–2019). All heat illness patients (i.e., hospitalized and non-hospitalized) were registered during 2010–2012, whereas only hospitalized patients were registered during 2014–2019. In each participating hospital, the emergency department physicians diagnosed heat illnesses, based on symptoms (e.g., high body temperature and signs of dehydration such as dizziness, myalgia, headache) and history of exposure to hot environments before the symptom onset [27, 28]. Patients with an infectious disease were excluded; i.e., we excluded cases diagnosed as infections because of the presence of bacteria in blood cultures or pneumonia on radiographs or computed tomography scans. The physicians collected the patient data from the medical records and registered them by using a web-based data collection system. Data were collected on variables such as symptom onset situation (e.g., sports, labor, or everyday life); parameters at hospital arrival such as vital signs, core body temperature (i.e., bladder or rectal temperature), and laboratory parameters (indicating liver, hepatic, and coagulation functions); cooling methods; and in-hospital deaths. Prognoses after discharge from the hospital emergency department were not investigated.

### Study participants

**Fig 1:**
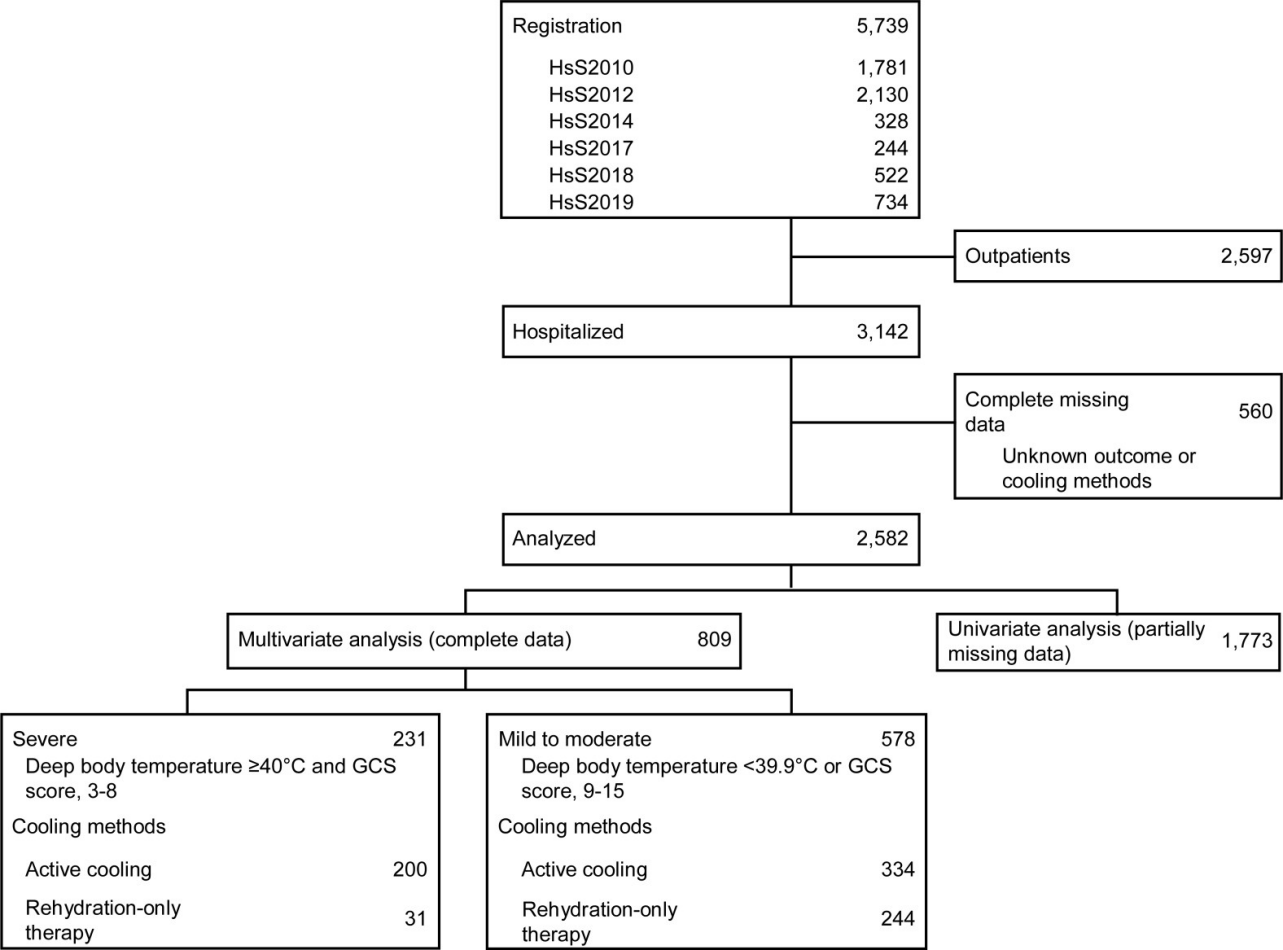
Patient selection process. Active cooling includes exclusively external, exclusively internal, and combined cooling. Rehydration-only therapy refers to fluid replacement without active cooling. HsS: Heat stroke Study; GCS: Glasgow Coma Scale.

The study participants were hospitalized patients diagnosed with heat stroke or heat exhaustion and registered in the HsS in 2010, 2012, 2014, 2017, 2018, and 2019. Of the 5739 registered patients, 3142 hospitalized patients with exceptionally low mortality risk; 560 patients with missing outcome or cooling methods data were excluded. Of the 2582 patients analyzed, 1773 patients with partially missing data underwent univariate analyses and 809 patients with complete data underwent multivariate analysis (Fig 1). We categorized the participants into the severe group (i.e., core body temperature, ≥40.0°C [313.15 K]; Glasgow Coma Scale [GCS] score, ≤8) or the mild-to-moderate group (i.e., all remaining patients), following Bouchama’s severity classification based on the core body temperature and central nervous system abnormalities [29].

### Endpoint

The endpoint was in-hospital mortality.

### Cooling methods

We classified cooling methods as active cooling therapy or rehydration-only therapy. The former included internal, external, and combined cooling; all patients who were treated with active cooling were also treated with rehydration therapy. Various active cooling methods used in the participating facilities were categorized as follows: external cooling included the cooling of body surfaces through cold-water immersion, evaporative and convective cooling, and body-cooling units; internal cooling included cooling of the body cavity through gastric lavage and bladder irrigation with cold water and intravascular temperature management by saline circulation through catheter balloons or extracorporeal circulation; and combined cooling included combinations of internal and external cooling methods. Rehydration-only therapy comprised intravenous fluid replacement without active cooling, in which extracellular fluid (lactate Ringer’s solution, acetate Ringer’s solution, etc.) at room temperature was usually used. Although some facilities used a refrigerator-cooled solution, we did not consider such therapy as an active cooling method because the fluid was not kept cool during infusion. We did not collect information on fluid temperature.

This classification of cooling methods is based on Bouchama’s theory of heat transmission in the human body [29]. Excess heat of the body core is transmitted to the body surface through the blood and by direct heat conduction. Furthermore, vasodilation increases blood flow to the skin. Heat is thereafter released into the atmosphere through the skin, usually by sweat evaporation. Cooling methods can apply to each of these processes. External cooling facilitates heat exchange through the body’s surfaces and internal cooling through the body’s core. Fluid replacement may enhance heat transfer from the core to the surface.

### Variables

We classified age as <5 years, 15–44 years, 45–64 years, 65–74 years, and ≥75 years; core body temperature as <39.0°C (<312.15 K), 39.0°C–39.9°C (312.15–313.05 K), 40.0–40.9°C (313.15–314.05 K), 41.0–41.9°C (314.15–315.05 K), and ≥42.0°C (≥315.15 K); GCS score as 3–5, 6–8, 9–14, and 15; onset situation as exertional (e.g., sports and labor) and non-exertional (e.g., everyday life); and year as 2010–2012 and 2014–2018. We defined liver damage, based on an aspartate transaminase level of ≥30 U/L (0.5 μkat/L) or an alanine aminotransferase level of ≥42 U/L (men: 0.7 μkat/L)/≥) or ≥23 U/L (women: 0.38 μkat/L); renal dysfunction based on a creatinine level of ≥1.07 mg/dL (men: 94.61 μmol/L)/≥) or ≥0.80 mg/dL (women: 70.74 μmol/L); and DIC based on a score ≥4 (Japanese Association for Acute Medicine scoring) [30]. This DIC score reflects DIC severity ranging from 0 (mild) to 6 (severe), depending on the presence of systemic inflammatory response syndrome, thrombocytopenia, prothrombin time international normalized ratio prolongation, and D-dimer increase.

### Data analysis

We stratified the patients with complete data into the “severe” group or the “mild-to-moderate” group and analyzed the group-wise association between cooling methods and in-hospital deaths. We conducted logistic regression analysis with in-hospital death as the dependent variable and the cooling method as the main independent variable. We also conducted a multivariate analysis to adjust for the following covariates that are likely to be associated with the prognoses of heat illness patients: sex, age, onset situation (i.e., exertional or non-exertional), core body temperature, liver damage, renal dysfunction, and DIC [31–34]. We also included the years when the HsS was conducted to consider unmeasured environmental, clinical, or meteorological changes. The variables were dichotomized before logistic regression analyses as follows: (1) cooling methods: active cooling, rehydration-only therapy; (2) sex: female, male; (3) age (years): ≤64, ≥65; (4) years: 2010–2014, 2017–2019; (5) onset situation: exertional, non-exertional; (6) core body temperature: <40.9°C and ≥41.0°C; (7) GCS score: 3–5 and 6–15; (8) liver damage: absent or present; (9) renal dysfunction: absent, present; and (10) DIC score ≥4 or ≤4. For each variable, we calculated the crude odds ratio in the univariate analysis and adjusted odds ratio (aOR) in the multivariate analysis, along with their respective 95% confidence intervals (CIs). The multivariate model included all of the abovementioned independent variables. We used SPSS Statistics version 25 (IBM, Armonk, NY) for the data analysis.

The sample size was determined in advance using the number of patients registered in the database. Therefore, we calculated the minimum detectable effect size for the given sample. The odds ratio needed to be 3.21 or higher to achieve statistical significance based on the given sample size of the severe cases (n = 231), the proportion of patients receiving rehydration-only therapy (13.4%), a 5% probability of type one error, a power of 0.8, and an assumption that the mortality rate among severe patients treated with active cooling methods was 20%. We used G*power ver.3.1.9.2 for this calculation [35].

We conducted univariate analyses among patients with missing data (S1 Fig). We excluded patients with unknown cooling methods and missing outcomes and classified the remaining patients into four groups: (1) patients with complete data of core body temperature ≥40.0°C (≥313.15 K) and GCS 3–8 (i.e., severe); (2) patients with incomplete severity data but who were likely to be classified as severe, based on the core body temperature (i.e., ≥40.0°C [≥313.15 It]) or GCS of 3–8 (i.e., likely to be severe); (3) patients with a core body temperature of <40.0°C (<313.15 K) or GCS 9–15 (i.e., mild-to-moderate); and (4) patients with completely missing severity data (i.e., unknown severity; S1 Fig). We thereafter compared the group-wise mortality rates.

## Results

Of the 809 patients analyzed for this study, 231 patients were categorized as “severe” and 578 patients as “mild-to-moderate.” Most patients received active cooling therapy (Table 1). Among all participants, men and individuals aged ≥65 years accounted for 69% and 61%, respectively. Non-exertional mechanisms were seen in 66% patients. Some mild-to-moderate patients had severe symptoms: 18% had a high body temperature (≥40°C), 15% had a GCS score ≤8, 64% had liver damage, and 77% had renal dysfunction. DIC and in-hospital death occurred in 28% and 15%, respectively, of the “severe” patients, and in 23% and 5%, respectively, of the “mild-to-moderate” patients. Among the severe cases, rehydration-only therapy was mostly provided to individuals aged ≥65 years. No apparent differences existed in the characteristics between therapies provided to patients in the “severe” and “likely to be severe” categories.

**Table 1:** Distribution of factors potentially associated with patient prognosis after heat illness onset.

	Cooling method			
	Severe group		Mild-to-moderate group	
	Active cooling (n = 200) ^a^	Rehydration-only therapy (n = 31) ^b^	Active cooling (n = 334) ^a^	Rehydration-only therapy (n = 244) ^b^
	n (%)	n (%)	n (%)	n (%)
**In-hospital deaths, n (%)**	43 (21.5)	11 (35.5)	13 (3.9)	14 (5.7)
**Cooling method, n (%)**				
**Exclusively external cooling**	91 (45.5)	0 (0.0)	242 (72.5)	0 (0.0)
**Exclusively internal cooling**	13 (6.5)	0 (0.0)	17 (5.1)	0 (0.0)
**Combined cooling**	96 (48.0)	0 (0.0)	75 (22.5)	0 (0.0)
**Rehydration-only therapy**	0 (0.0)	31 (100.0)	0 (0.0)	244 (100.0)
**Male patient, n (%)**	142 (71.0)	16 (51.6)	229 (68.6)	172 (70.5)
**Age (y), n (%)**				
**0–14**	0 (0.0)	0 (0.0)	5 (1.5)	3 (1.2)
**15–44**	27 (13.5)	2 (6.5)	44 (13.2)	44 (18.0)
**45–64**	67 (33.5)	2 (6.5)	71 (21.3)	52 (21.3)
**65–74**	37 (18.5)	12 (38.7)	60 (18.0)	44 (18.0)
**≥75**	69 (34.5)	15 (48.4)	154 (46.1)	101 (41.4)
**Study year, n (%)** ^**c**^				
**2010**	69 (34.5)	1 (3.2)	94 (28.1)	43 (17.6)
**2012**	20 (10.0)	3 (9.7)	25 (7.5)	24 (9.8)
**2014**	14 (7.0)	7 (22.6)	49 (14.7)	16 (6.6)
**2017**	8 (4.0)	0 (0.0)	22 (6.6)	34 (13.9)
**2018**	33 (16.5)	8 (25.8)	76 (22.8)	42 (17.2)
**2019**	56 (28.0)	12 (38.7)	68 (20.4)	85 (34.8)
**Onset situation, n (%)** ^**d**^				
**Non-exertional**	133 (66.5)	25 (80.6)	227 (68.0)	148 (60.7)
**Exertional**	67 (33.5)	6 (19.4)	107 (32.0)	96 (39.3)
**Core body temperature (°C), n (%)**				
**42.0+**	55 (27.5)	5 (16.1)	5 (1.5)	0 (0.0)
**41.0–41.9**	85 (42.5)	13 (41.9)	20 (16.0)	4 (1.6)
**40.0–40.9**	60 (30.0)	13 (41.10)	58 (17.4)	19 (7.8)
**39.0–39.9**	0 (0.0)	0 (0.0)	119 (35.6)	45 (18.4)
**<38.9**	0 (0.0)	0 (0.0)	32 (39.5)	176 (72.1)
**Glasgow Coma Scale score, n (%)**				
**3–5**	151 (75.5)	21 (67.7)	37 (11.1)	14 (5.7)
**6–8**	49 (24.5)	10 (32.3)	26 (7.8)	12 (4.9)
**9–14**	0 (0.0)	0 (0.0)	217 (65.0)	133 (54.5)
**15**	0 (0.0)	0 (0.0)	54 (16.2)	85 (34.8)
**Liver damage, number (%)** ^**e**^				
**Present**	169 (84.5)	25 (80.6)	221 (66.2)	148 (60.7)
**Renal dysfunction, number (%)** ^**f**^				
**Present**	180 (90.0)	27 (87.1)	261 (78.1)	186 (76.2)
**DIC, n (%)** ^**g**^				
**DIC score ≥4**	57 (28.5)	8 (25.8)	49 (14.7)	37(15.2)

^a^ Includes exclusively external, exclusively internal, and combined cooling. External cooling is the cooling of body surfaces through cold-water immersion, evaporative plus convective cooling, and body-cooling units. Internal cooling is the cooling of the body cavity through gastric lavage and bladder irrigation with ice water, intravascular ice cradle, and temperature management by extracorporeal membrane oxygenation. Combined cooling is the combination of internal and external cooling methods.

^b^ Fluid replacement without active cooling.

^c^ Indicates the year when the Heat stroke Study was conducted.

^d^ Non-exertional onset condition is the onset of heat illness during participation in daily activities; exertional onset condition is the onset of heat illness during participation in sports and labor.

^e^ Damage, as indicated by an aspartate transaminase level of ≥30 U/L (0.5 μkat/L) or an alanine aminotransferase level of ≥42 U/L (men: 0.7 μkat/L) or ≥23 U/L (women: 0.38 μkat/L).

^f^ Dysfunction, as indicated by a creatinine level of ≥1.07 mg/dL (men: 94.61 μmol/L) or ≥0.80 mg/dL (women: 70.74 μmol/L).

^g^ Disseminated intravascular coagulation (DIC), defined as a score ≥4, based on the Japanese Association for Acute Medicine scoring system.

Among the severe cases, rehydration-only therapy was significantly associated with higher in-hospital mortality (aOR, 3.29; 95% CI: 1.21–8.90). Among the mild-to-moderate patients, cooling methods were not associated with lower mortality (aOR, 2.32; 95% CI: 0.92–5.84). Lower GCS scores and the presence of DIC were associated with an increased risk of in-hospital deaths among the “severe” and “likely to be severe” cases. The presence of liver damage was associated with a higher in-hospital mortality only in the severe cases (Table 2).

**Table 2:** Odds ratios of factors potentially associated with patient prognosis after heat illness onset.

		Heat stroke (severe)		Heat exhaustion (mild-to-moderate)	
		(n = 231)		(n = 578)	
		cOR (95% CI)	aOR (95% CI)	cOR (95% CI)	aOR (95% CI)
**Cooling methods (ref: active cooling)** ^**a**^					
	**Rehydration-only therapy** ^**b**^	2.01 (0.89–4.51)	3.29 (1.21–8.90)	1.50 (0.69–3.26)	2.32 (0.92–5.84)
**Sex (ref: female)**					
	**Male**	0.90 (0.47–1.73)	1.34 (0.60–2.99)	0.53 (0.25–1.17)	0.80 (0.31–2.11)
**Age (ref: ≤64)**					
	**≥65 y**	1.10 (0.59–2.03)	0.74 (0.32–1.69)	2.80 (1.04–7.49)	2.45 (0.76–7.92)
**Study year (ref: 2017–2019)** ^**c**^					
	**2010–2014**	0.58 (0.31–1.07)	0.53 (0.26–1.07)	0.98 (0.89–1.09)	1.76 (0.72–4.30)
**Onset situation (ref: exertional)** ^**d**^					
	**Non-exertional**	1.43 (0.72–2.83)	1.27 (0.54–2.96)	3.25 (1.11–9.53)	2.01 (0.56–7.14)
**Core body temperature (ref: <40.9°C)**					
	**≥41.0°C**	1.84 (0.90–3.75)	1.48 (0.67–3.29)	0.00 (0.00-)	0.00 (0.00-)
**Glasgow Coma Scale score (ref: 6–15)**					
	**3–5**	4.30 (1.62–11.40)	4.76 (1.64–13.78)	14.96 (6.55–34.15)	15.56 (5.90–41.04)
**Liver damage (ref: absent)** ^**e**^					
	**Present**	4.04 (1.19–13.73)	3.09 (0.85–11.24)	4.78 (1.42–16.06)	3.36 (0.92–12.31)
**Renal dysfunction (ref: absent)** ^**f**^					
	**Present**	3.69 (0.84–16.23)	3.28 (0.68–15.75)	2.42 (0.71–8.17)	1.03 (0.27–3.92)
**DIC (ref: DIC score ≤4)** ^**g**^					
	**DIC score ≥4**	3.29 (1.73–6.24)	4.17 (1.96–8.87)	5.16 (2.32–11.45)	3.39 (1.34–8.59)

All variables were dichotomized before single variable and multivariable logistic analyses.

cOR: crude odds ratio (univariate analysis), aOR: adjusted odds ratio (multivariable analysis).

^a^ Includes exclusively external, exclusively internal, and combined cooling.

^b^ Fluid replacement without active cooling.

^c^ Year when the Heat stroke Study was conducted.

^d^ Non-exertional onset situation is the onset of heat illness during participation in daily life activities. Exertional onset situation is the onset of heat illness during participation in sports and labor.

^e^ Damage, as indicated by an aspartate transaminase level of ≥30 U/L (0.5 μkat/L) or alanine aminotransferase level of ≥42 U/L (men: 0.7 μkat/L) or ≥23 U/L (women: 0.38 μkat/L).

^f^ Dysfunction, as indicated by a creatinine level of ≥1.07 mg/dL (men: 94.61 μmol/L) or ≥0.80 mg/dL (women: 70.74 μmol/L).

^g^ Disseminated intravascular coagulation (DIC), defined as a score ≥4, based on the Japanese Association for Acute Medicine scoring system.

Among 1773 patients with partial missing data, 102 patients were categorized as “severe;” 161 patients, as “likely to be severe;” 1405 patients, as “mild-to-moderate;” and 105 patients, as “unknown severity” (S1 Table). In-hospital death rates were higher for “severe” and “likely to be severe” patients who received rehydration-only therapy than for those who received active cooling: 33% vs. 22%, respectively, and 29% vs. 23%, respectively. These rates were lower among “mild-to-moderate” patients (1% vs. 4%) and “unknown severity” patients (0% vs. 14%). S2 Table presents the results of the univariate analysis. S3 Table presents the characteristics of the excluded patients with missing data; the findings did not differ from those of the included patients.

## Discussion

This study compared the prognosis of heat stroke and heat exhaustion patients treated with active cooling methods or with rehydration-only therapy using data from a nationwide heat stroke and heat exhaustion registry database. This study showed that, after adjusting for potential covariates, patients with severe cases of heat illnesses in which active cooling was administered were more likely to survive hospitalization than those who received rehydration-only therapy. Our findings suggest that active cooling is important to improve the outcomes of severe heat illness patients. Rehydration is an important component of heat illness management; however, it may not provide sufficient benefit to severe cases without concurrent active cooling.

By contrast, active cooling was not associated with lower mortality among patients with mild-to-moderate cases. This finding does not imply that active cooling is useless in cases of heat exhaustion. Mortality rates among mild-to-moderate cases may be too low to detect differences. Other prognostic variables such as neurological complications could more appropriately be used to evaluate the treatment effects. However, they are not available in the database.

The large scale of this study allowed us to evaluate the benefits of active cooling in heat illness patients by using the patients’ prognosis as the endpoint rather than by using a proxy measure. Severe heat stroke that results in death is a rare condition; therefore, single-center studies cannot include a sufficient number of severe cases to evaluate treatment effects on mortality. Most previous studies have consequently used proxy measures such as the speed of lowering the core body temperature [36] to evaluate treatment effects among mild-to-moderate cases (i.e., heat exhaustion) that had mostly no severe outcomes (i.e., mortality or complications) [12].

In addition, we determined the associations between active cooling and patients with heat illness and compared these with those of patients who received rehydration-only therapy. Active cooling is the standard treatment for severe heat stroke; therefore, a small proportion of patients receive rehydration-only therapy [29]. Our multicenter study included a sufficient number of these patients to allow comparisons.

We did not explore why some patients, particularly patients in the “severe” group, did not receive active cooling, although it is the standard therapy. A possibility is that emergency physicians who prescribed rehydration-only therapy may have overestimated its effects of enhancing blood flow for heat exchange [37]. An alternative reason is that they may have hesitated using active cooling because of peripheral vasoconstriction and shivering, which may delay surface heat exchange and temperature reduction [38]. However, we observed the insufficiency of rehydration-only therapy to improve patients’ prognosis. We should suggest that guidelines strongly recommend the application of active cooling methods to heat illness patients.

This study has several limitations. First, compared to interventional studies, the observational nature of the study limits the inference of causality. Nonetheless, ethical concerns prevent a study design in which patients are assigned rehydration-only therapy instead of the standard treatment (i.e., active cooling). Second, we were unable to collect standardized data on the influence of cooling speed, target temperature, and different types of external cooling methods because of interinstitutional differences and lack of specific guidelines regarding active cooling methods. Therefore, some patients may have received active cooling at a slower than recommended speed [38, 39]. This factor could have reduced the improvement of active cooling patients’ outcomes, but we were able to demonstrate its effectiveness. Therefore, this limitation is unlikely to affect the results of this study.

Third, we did not collect data on the treatment of injured organs after body cooling. We assumed that timely body cooling could prevent organ injuries; moreover, no recommended treatments for organ injuries after active cooling among heat stroke patients exist [11]. Therefore, non-adjustment for treatments in the multivariate model was unlikely to distort our results.

Finally, we excluded many patients because of missing data. Among these, “severe” or “near-severe” patients had increased in-hospital mortality when receiving rehydration-only therapy, whereas “mild-to-moderate” patients had not increased mortality when receiving rehydration-only therapy. The severity, outcome, or applied cooling method were unknown in a few individuals, but these lacking data did not affect the results. If these patients had been included in the main analysis without the missing data, the efficacy of active cooling among severe cases would have remained unchanged.

## Conclusions

Active cooling was associated with lower in-hospital mortality among severe cases of heat illness than was rehydration-only therapy; however, this finding did not occur for mild-to-moderate cases of heat illness. Our results support the recommendation of applying active cooling to reduce the core body temperature in addition to rehydration therapy for heatstroke patients.

## Supporting information

S1 Fig Selection process of patients with partially missing data. Active cooling includes exclusively external, exclusively internal, and combined cooling. Rehydration-only therapy refers to fluid replacement alone without active cooling. GCS: Glasgow Coma Scale.
(DOCX)

S1 Table Outcomes and characteristics of patients (n = 1773) with partially missing data. (DOCX)

S2 Table Crude odds ratios of factors potentially associated with the prognoses of patients with partially missing data. (DOCX)

S3 Table Outcomes and characteristics of patients with completely missing data (n = 560). (DOCX)

S1 Text Participating hospitals and data collection periods. (DOCX)
