# Supplementary material for: Association between active cooling and lower mortality among patients with heat stroke and heat exhaustion
Source: PLoS One. 2021 Nov 17;16(11):e0259441. doi: 10.1371/journal.pone.0259441 (PMC8598059; doi:10.1371/journal.pone.0259441)
Supplement: S1 Fig — Active cooling includes exclusively external, exclusively internal, and combined cooling. Rehydration-only therapy refers to fluid replacement alone without active cooling. GCS: Glasgow Coma Scale. (DOCX) [file pone.0259441.s001.docx]

**S1** **Fig.** Selection process of patients with partially missing data


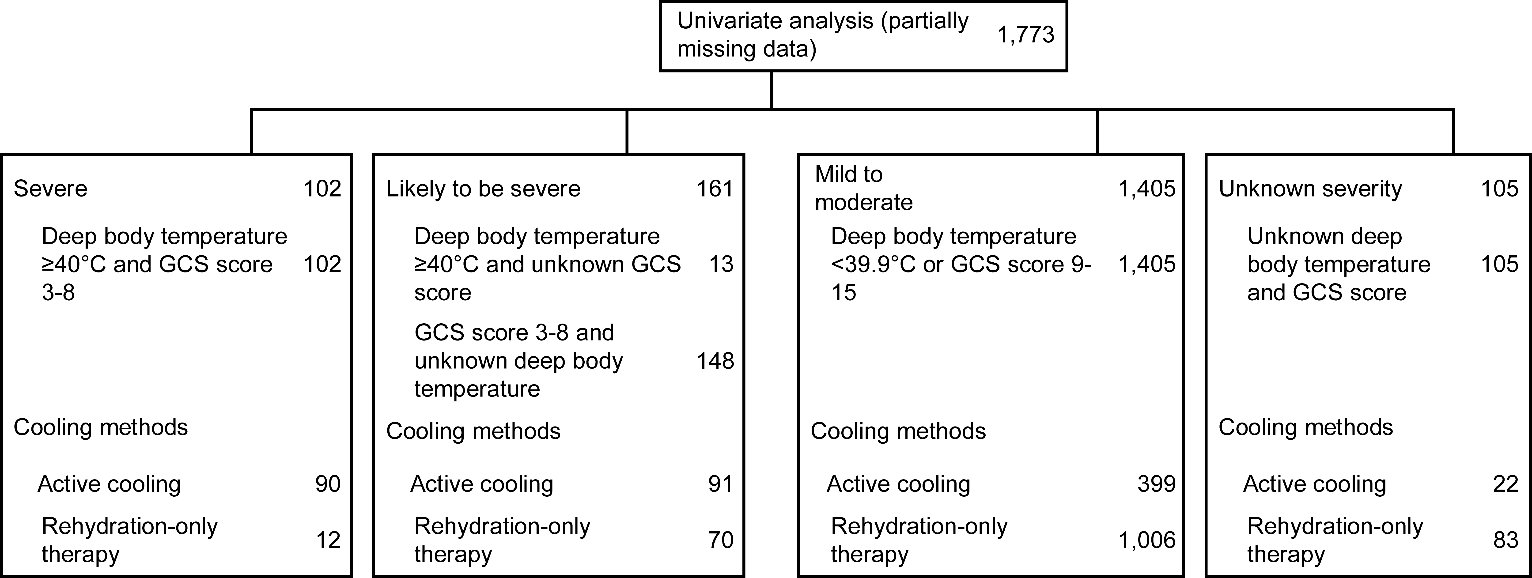


Active cooling includes exclusively external, exclusively internal, and combined cooling; rehydration-only therapy refers to fluid replacement alone without active cooling

GCS: Glasgow Coma Scale.
