# Supplementary material for: Association between active cooling and lower mortality among patients with heat stroke and heat exhaustion
Source: PLoS One. 2021 Nov 17;16(11):e0259441. doi: 10.1371/journal.pone.0259441 (PMC8598059; doi:10.1371/journal.pone.0259441)
Supplement: S1 Table — (DOCX) [file pone.0259441.s002.docx]

**S1 Table. Outcomes and characteristics of patients with partially missing data (n=1773).**

|  |  | Cooling methods | | | | | | | | | | | | | | | | | | |
| --- | --- | --- | --- | --- | --- | --- | --- | --- | --- | --- | --- | --- | --- | --- | --- | --- | --- | --- | --- | --- |
|  |  | Severe (n=102) | | | |  | Likely to be severe (n=161) | | | |  | Mild to moderate (n=1405) | | | |  | Unknown severity (n=105) | | | |
|  | | Active cooling^a^ (n=90) | | Rehydration-only therapy^b^ (n=12) | |  | Active cooling^a^ (n=91) | | Rehydration-only therapy^b^ (n=70) | |  | Active cooling^a^ (n=399) | | Rehydration-only therapy^b^ (n=1006) | |  | Active cooling^a^ (n=22) | | Rehydration-only therapy^b^ (n=83) | |
|  |  | n (%) |  | n (%) |  |  | n (%) |  | n (%) |  |  | n (%) |  | n (%) |  |  | n (%) |  | n (%) |  |
| In-hospital deaths, number (%) | | 20 (22.2) |  | 4 (33.3) |  |  | 21 (23.1) |  | 20 (28.6) |  |  | 15 (3.8) |  | 13 (1.3) |  |  | 3 (13.6) |  | 0 (0.0) |  |
| Cooling methods, number (%) | |  |  |  |  |  |  |  |  |  |  |  |  |  |  |  |  |  |  |  |
|  | Exclusively external cooling | 37 (41.1) |  | 0 (0.0) |  |  | 55 (60.4) |  | 0 (0.0) |  |  | 311 (77.9) |  | 0 (0.0) |  |  | 19 (86.4) |  | 0 (0.0) |  |
|  | Exclusively internal cooling | 7 (7.8) |  | 0 (0.0) |  |  | 6 (6.6) |  | 0 (0.0) |  |  | 21 (5.3) |  | 0 (0.0) |  |  | 1 (4.5) |  | 0 (0.0) |  |
|  | Combined cooling | 46 (51.1) |  | 0 (0.0) |  |  | 30 (33.0) |  | 0 (0.0) |  |  | 67 (16.8) |  | 0 (0.0) |  |  | 2 (9.1) |  | 0 (0.0) |  |
|  | Rehydration-only therapy | 0 (0.0) |  | 12 (100.0) |  |  | 0 (0.0) |  | 70 (100.0) |  |  | 0 (0.0) |  | 1,006 (100.0) |  |  |  |  | 83 (100.0) |  |
| Male, number (%) | | 52 (58.4) |  | 9 (75.0) |  |  | 57 (62.6) |  | 44 (62.9) |  |  | 274 (68.8) |  | 749 (74.8) |  |  | 14 (66.7) |  | 51 (63.0) |  |
|  | Unknown | 1 |  | 0 |  |  | 0 |  | 0 |  |  | 1 |  | 4 |  |  | 1 |  | 2 |  |
| Age (years), number (%) | |  |  |  |  |  |  |  |  |  |  |  |  |  |  |  |  |  |  |  |
|  | 0-14 | 0 (0.0) |  | 0 (0.0) |  |  | 1 (1.1) |  | 1 (1.4) |  |  | 15 (3.8) |  | 37 (3.7) |  |  | 1 (4.5) |  | 9 (10.8) |  |
|  | 15-44 | 9 (10.1) |  | 1 (8.3) |  |  | 12 (13.2) |  | 7 (10.0) |  |  | 83 (20.9) |  | 266 (26.5) |  |  | 4 (18.2) |  | 20 (24.1) |  |
|  | 45-64 | 25 (28.1) |  | 2 (16.7) |  |  | 28 (30.8) |  | 12 (17.1) |  |  | 72 (18.1) |  | 227 (22.6) |  |  | 7 (31.8) |  | 18 (21.7) |  |
|  | 65-74 | 19 (21.3) |  | 2 (16.7) |  |  | 12 (13.2) |  | 12 (17.1) |  |  | 70 (17.6) |  | 141 (14.1) |  |  | 1 (4.5) |  | 14 (16.9) |  |
|  | ≥75 | 36 (40.4) |  | 7 (58.3) |  |  | 38 (41.8) |  | 38 (54.3) |  |  | 158 (39.7) |  | 332 (33.1) |  |  | 9 (40.9) |  | 22 (26.5) |  |
|  | Unknown | 1 |  | 0 |  |  | 0 |  | 0 |  |  | 1 |  | 3 |  |  | 0 |  | 0 |  |
| Year^c^, number (%) | |  |  |  |  |  |  |  |  |  |  |  |  |  |  |  |  |  |  |  |
|  | 2010 | 43 (47.8) |  | 3 (25.0) |  |  | 38 (41.8) |  | 14 (20.0) |  |  | 119 (29.8) |  | 206 (20.5) |  |  | 6 (27.3) |  | 20 (24.1) |  |
|  | 2012 | 18 (20.0) |  | 5 (41.7) |  |  | 21 (23.1) |  | 16 (22.9) |  |  | 111 (27.8) |  | 201 (20.0) |  |  | 14 (63.6) |  | 47 (56.6) |  |
|  | 2014 | 5 (5.6) |  | 1 (8.3) |  |  | 5 (5.5) |  | 1 (1.4) |  |  | 58 (14.5) |  | 28 (2.8) |  |  | 0 (0.0) |  | 0 (0.0) |  |
|  | 2017 | 2 (2.2) |  | 0 (0.0) |  |  | 5 (5.5) |  | 5 (7.1) |  |  | 20 (5.0) |  | 94 (9.3) |  |  | 1 (4.5) |  | 7 (8.4) |  |
|  | 2018 | 3 (3.3) |  | 0 (0.0) |  |  | 8 (8.8) |  | 15 (21.4) |  |  | 26 (6.5) |  | 216 (21.5) |  |  | 0 (0.0) |  | 2 (2.4) |  |
|  | 2019 | 19 (21.1) |  | 3 (25.0) |  |  | 14 (15.4) |  | 19 (27.1) |  |  | 65 (16.3) |  | 261 (25.9) |  |  | 1 (4.5) |  | 7 (8.4) |  |
|  | Unknown | 0 |  | 0 |  |  | 0 |  | 0 |  |  | 0 |  | 0 |  |  | 0 |  | 0 |  |
| Onset situation^d^, number (%) | |  |  |  |  |  |  |  |  |  |  |  |  |  |  |  |  |  |  |  |
|  | Non-exertional | 59 (79.7) |  | 6 (85.7) |  |  | 52 (65.8) |  | 52 (80.0) |  |  | 210 (58.5) |  | 438 (45.6) |  |  | 11 (57.9) |  | 32 (43.8) |  |
|  | Exertional | 15 (20.3) |  | 1 (14.3) |  |  | 27 (34.2) |  | 13 (20.0) |  |  | 149 (41.5) |  | 522 (54.4) |  |  | 8 (42.1) |  | 41 (56.2) |  |
|  | Unknown | 16 |  | 5 |  |  | 12 |  | 5 |  |  | 40 |  | 46 |  |  | 3 |  | 10 |  |
| Deep temperature (°C), number (%) | |  |  |  |  |  |  |  |  |  |  |  |  |  |  |  |  |  |  |  |
|  | ≥42.0 | 19 (21.1) |  | 5 (41.7) |  |  | 1 (8.3) |  | 0 (0.0) |  |  | 2 (1.2) |  | 0 (0.0) |  |  | 0 (0.0) |  | 0 (0.0) |  |
|  | 41.0-41.9 | 36 (41.0) |  | 5 (41.7) |  |  | 5 (41.7) |  | 0 (0.0) |  |  | 11 (6.4) |  | 2 (1.6) |  |  | 0 (0.0) |  | 0 (0.0) |  |
|  | 40.0-40.9 | 49 (38.9) |  | 2 (16.7) |  |  | 6 (50.0) |  | 1 (100.0) |  |  | 29 (17.0) |  | 15 (11.6) |  |  | 0 (0.0) |  | 0 (0.0) |  |
|  | 39.0-39.9 | 0 (0.0) |  | 0 (0.0) |  |  | 0 (0.0) |  | 0 (0.0) |  |  | 56 (32.7) |  | 27 (20.9) |  |  | 0 (0.0) |  | 0 (0.0) |  |
|  | ≤38.9 | 0 (0.0) |  | 0 (0.0) |  |  | 0 (0.0) |  | 0 (0.0) |  |  | 73 (42.7) |  | 85 (65.9) |  |  | 0 (0.0) |  | 0 (0.0) |  |
|  | Unknown | 0 |  | 0 |  |  | 79 |  | 69 |  |  | 228 |  | 877 |  |  | 22 |  | 83 |  |
| Glasgow Coma Scale score, number (%) | |  |  |  |  |  |  |  |  |  |  |  |  |  |  |  |  |  |  |  |
|  | 3-5 | 87 (69.0) |  | 9 (69.2) |  |  | 46 (58.2) |  | 46 (66.7) |  |  | 20 (5.1) |  | 11 (1.1) |  |  | 0 (0.0) |  | 0 (0.0) |  |
|  | 6-8 | 39 (31.0) |  | 4 (30.8) |  |  | 33 (41.8) |  | 23 (33.3) |  |  | 18 (4.8) |  | 9 (0.9) |  |  | 0 (0.0) |  | 0 (0.0) |  |
|  | 9-14 | 0 (0.0) |  | 0 (0.0) |  |  | 0 (0.0) |  | 0 (0.0) |  |  | 210 (53.3) |  | 324 (32.4) |  |  | 0 (0.0) |  | 0 (0.0) |  |
|  | 15 | 0 (0.0) |  | 0 (0.0) |  |  | 0 (0.0) |  | 0 (0.0) |  |  | 146 (37.1) |  | 655 (65.6) |  |  | 0 (0.0) |  | 0 (0.0) |  |
|  | Unknown | 0 |  | 0 |  |  | 12 |  | 1 |  |  | 5 |  | 7 |  |  | 22 |  | 83 |  |
| Liver damage^e^, number (%) | |  |  |  |  |  |  |  |  |  |  |  |  |  |  |  |  |  |  |  |
|  | Present liver damage | 71 (81.6) |  | 11 (91.7) |  |  | 71 (82.6) |  | 49 (75.4) |  |  | 242 (66.7) |  | 561 (58.9) |  |  | 12 (66.7) |  | 38 (54.3) |  |
|  | Unknown | 3 |  | 0 |  |  | 5 |  | 5 |  |  | 36 |  | 53 |  |  | 4 |  | 13 |  |
| Renal dysfunction^f^, number (%) | |  |  |  |  |  |  |  |  |  |  |  |  |  |  |  |  |  |  |  |
|  | Present renal disfunction | 70 (89.7) |  | 11 (100.0) |  |  | 75 (89.3) |  | 53 (84.1) |  |  | 237 (76.7) |  | 697 (76.3) |  |  | 10 (66.7) |  | 45 (68.2) |  |
|  | Unknown | 12 |  | 0 |  |  | 7 |  | 7 |  |  | 90 |  | 93 |  |  | 7 |  | 17 |  |
| DIC^g^, number (%) | |  |  |  |  |  |  |  |  |  |  |  |  |  |  |  |  |  |  |  |
|  | Present DIC | 9 (36.0) |  | 2 (50.0) |  |  | 21 (30.9) |  | 20 (47.6) |  |  | 33 (16.1) |  | 41 (7.6) |  |  | 5 (38.5) |  | 3 (8.8) |  |
|  | Unknown | 65 |  | 8 |  |  | 23 |  | 28 |  |  | 194 |  | 470 |  |  | 9 |  | 49 |  |

^a^ Includes exclusively external, exclusively internal, and combined cooling. External cooling is the cooling of body surfaces through cold-water immersion, evaporative plus convective cooling, and body-cooling units. Internal cooling is the cooling of the body cavity through gastric lavage and bladder irrigation with ice water, intravascular ice cradle, and temperature management by extracorporeal membrane oxygenation. Combined cooling is the combination of internal and external cooling methods

^b^ Fluid replacement without active cooling

^c^ Year when the Heatstroke STUDY was performed

^d^ Non-exertional: onset of heat illness during participation in daily activities; exertional: onset of heat illness during participation in sports and labor

^e^ Damage indicated by aspartate transaminase levels ≥30 U/L (0.5 µkat/L) or alanine aminotransferase levels ≥42 U/L (0.7 µkat/L; male) or ≥23 U/L (0.38 µkat/L; female)

^f^ Dysfunction indicated by creatinine levels ≥1.07 mg/dL (94.61 µmol/L; male) or ≥0.80 mg/dL (70.74 µmol/L; female)

^g^ Disseminated Intravascular Coagulation (DIC) defined by a score ≥4 according to the Japanese Association for Acute Medicine scoring system
