# Supplementary material for: Association between active cooling and lower mortality among patients with heat stroke and heat exhaustion
Source: PLoS One. 2021 Nov 17;16(11):e0259441. doi: 10.1371/journal.pone.0259441 (PMC8598059; doi:10.1371/journal.pone.0259441)
Supplement: S2 Table — (DOCX) [file pone.0259441.s003.docx]

**S2 Table.** **Crude odds ratios of factors potentially associated with the prognoses of patients with partially missing data.**

|  |  | Severe (n=102) |  | Likely to be severe (n=161) |  | Mild to moderate (n=1405) |  | Unknown severity (n=105) |
| --- | --- | --- | --- | --- | --- | --- | --- | --- |
|  |  | cOR (95% CI) |  | cOR (95% CI) |  | cOR (95% CI) |  | cOR (95% CI) |
| Cooling methods (ref: active cooling^a^) | | | | | | |  |  |
|  | Rehydration-only therapy^b^ | 1.75 (0.48–6.42) |  | 1.33 (0.65–2.71) |  | 0.34 (0.16–0.71) |  | 0.00 (0.00–) |
| Sex (ref: female) | |  |  |  |  |  |  |  |
|  | Male | 0.81 (0.32–2.09) |  | 1.20 (0.57–2.52) |  | 0.77 (0.35–1.73) |  | 0.27 (0.02–3.12) |
| Age (ref: ≤64) | |  |  |  |  |  |  |  |
|  | ≥65 | 0.95 (0.37–2.46) |  | 1.95 (0.89–4.25) |  | 3.57 (1.43–8.90) |  | 112707547.2 (0.00–) |
| Year^c^ (ref: 2017–2019) | |  |  |  |  |  |  |  |
|  | 2010–2014 | 2.09 (0.64–6.80) |  | 1.28 (0.62–2.66) |  | 0.81 (0.39–1.72) |  | 57695533.89 (0.00–) |
| Onset situation^d^ (ref: exertional) | |  |  |  |  |  |  |  |
|  | Non-exertional | 1.66 (0.42–6.52) |  | 2.30 (0.87–6.04) |  | 6.14 (2.11–17.84) |  | 121160613.9 (0.00–) |
| Deep temperature (ref: ≤40.9°C) | |  |  |  |  |  |  |  |
|  | ≥41.0°C | 1.98 (0.71–5.54) |  | 1.20 (0.06–24.47) |  | 2.59 (0.54–12.46) |  | – |
| Glasgow Coma Scale score (ref: 6–15) | |  |  |  |  |  |  |  |
|  | 3–5 | 8.50 (1.87–38.68) |  | 3.73 (1.52–9.19) |  | 35.56 (14.67–98.16) |  | – |
| Liver damage^e^ (ref: absent) | |  |  |  |  |  |  |  |
|  | Present | 2.58 (0.54–12.24) |  | 748634771.3 (0.00–) |  | 17.13 (2.32–126.65) |  | 103115414.7 (0.00–) |
| Renal dysfunction^f^ (ref: absent) | |  |  |  |  |  |  |  |
|  | Present | 2.45 (0.28–21.11) |  | 7.32 (0.94–56.83) |  | 2.19 (0.65–7.38) |  | 29916200.8 (0.00–) |
| DIC^g^ (ref: DIC score ≤4) | |  |  |  |  |  |  |  |
|  | DIC score ≥4 | 923128532.0 (0.00–) |  | 2.46 (1.01–6.04) |  | 5.39 (1.54–18.86) |  | 230782120.4 (0.00–) |

Cases with missing data were excluded from the analysis. All variables were dichotomized prior to single variable and multivariable logistic analyses

cOR: crude odds ratio (univariate analysis), aOR: adjusted odds ratio (multivariable analysis)

^a^ Includes exclusively external, exclusively internal, and combined cooling

^b^ Fluid replacement without active cooling

^c^ Year when the Heatstroke STUDY was performed

^d^ Non-exertional: onset of heat illness during participation in daily life activities; exertional: onset of heat illness during participation in sports and labor

^e^ Damage indicated by aspartate transaminase levels ≥30 U/L (0.5 µkat/L) or alanine aminotransferase levels ≥42 U/L (0.7 µkat/L; male) or ≥23 U/L (0.38 µkat/L; female)

^f^ Dysfunction indicated by creatinine levels ≥1.07 mg/dL (94.61 µmol/L; male) or ≥0.80 mg/dL (70.74 µmol/L; female)

^g^ Disseminated Intravascular Coagulation (DIC) defined by a score ≥4 according to the Japanese Association for Acute Medicine scoring system
