# Supplementary material for: Association between active cooling and lower mortality among patients with heat stroke and heat exhaustion
Source: PLoS One. 2021 Nov 17;16(11):e0259441. doi: 10.1371/journal.pone.0259441 (PMC8598059; doi:10.1371/journal.pone.0259441)
Supplement: S3 Table — (DOCX) [file pone.0259441.s004.docx]

**S3 Table.** **Outcomes and characteristics of patients with completely missing data.** (n = 560)

|  |  | n (%) |
| --- | --- | --- |
| In-hospital deaths, number (%) | | 36 (17.5) |
|  | Unknown | 354 |
| Cooling methods^a^, number (%) | |  |
|  | Exclusively external cooling | 75 (36.6) |
|  | Exclusively internal cooling | 3 (1.5) |
|  | Combined cooling | 33 (16.1) |
|  | Rehydration-only therapy | 94 (45.9) |
|  | Unknown | 205 |
| Male, number (%) | | 383 (69.0) |
|  | Unknown | 5 |
| Age (years), number (%) | |  |
|  | 0–14 | 19 (3.4) |
|  | 15–44 | 111 (20.0) |
|  | 45–64 | 125 (22.6) |
|  | 65–74 | 86 (15.5) |
|  | ≥75 | 213 (38.4) |
|  | Unknown | 6 |
| Year^b^, number (%) | |  |
|  | 2010 | 75 (13.4) |
|  | 2012 | 78 (13.9) |
|  | 2014 | 144 (25.7) |
|  | 2017 | 46 (8.2) |
|  | 2018 | 93 (16.6) |
|  | 2019 | 124 (22.1) |
|  | Unknown | 0 |

^a^ Active cooling includes exclusively external, exclusively internal, and combined cooling. External cooling is the cooling of body surfaces through cold-water immersion, evaporative plus convective cooling, and body-cooling units. Internal cooling is the cooling of the body cavity through gastric lavage and bladder irrigation with ice water, intravascular ice cradle, and temperature management by extracorporeal membrane oxygenation. Combined cooling is the combination of internal and external cooling methods. Rehydration-only therapy refers to fluid replacement without active cooling

^b^ Year when the Heatstroke STUDY was performed
