# Supplementary material for: Association between active cooling and lower mortality among patients with heat stroke and heat exhaustion
Source: PLoS One. 2021 Nov 17;16(11):e0259441. doi: 10.1371/journal.pone.0259441 (PMC8598059; doi:10.1371/journal.pone.0259441)
Supplement: S1 Text — (DOCX) [file pone.0259441.s005.docx]

**S1 Text. Participating hospitals and data collection periods.**

| **Participating hospitals** | **Heat stroke STUDY** |
| --- | --- |
| Aizawa Hospital | 2010,12,14,17,18,19 |
| Daiyukai General Hospital | 2010,12,14,17,18,19 |
| Dokkyo Medical University Saitama Medical Center | 2010,12,14,17,18,19 |
| Hyogo Prefectural Kakogawa Medical Center | 2010,12,14,17,18,19 |
| Ishikawa Prefectural Central Hospital | 2010,12,14,17,18,19 |
| Japanese Red Cross Ishinomaki Hospital | 2010,12,14,17,18,19 |
| Japanese Red Cross Maebashi Hospital | 2010,12,14,17,18,19 |
| Japanese Red Cross Medical Center | 2010,12,14,17,18,19 |
| Kagawa Prefectural Central Hospital | 2010,12,14,17,18,19 |
| Nihon University Itabashi Hospital | 2010,12,14,17,18,19 |
| St. Luke's International Hospital | 2010,12,14,17,18,19 |
| Teikyo University Hospital | 2010,12,14,17,18,19 |
| Yamaguchi University Hospital | 2010,12,14,17,18,19 |
| Japanese Red Cross Akita Hospital | 2010,12,14,17,18 |
| Nippon Medical School Hospital | 2010,12,14,17,18 |
| Tokyo Medical University Hachioji Medical Center | 2010,12,14,17,18 |
| Mito Saiseikai General Hospital | 2010,12,14,18,19 |
| Osaka Mishima Emergency Critical Care Center | 2010,12,14,18,19 |
| Nihon University Hospital | 2010,12,17,18,19 |
| Hamamatsu Medical Center | 2010,14,17,18,19 |
| Kawasaki Municipal Hospital | 2010,14,17,18,19 |
| Ome Municipal General Hospital | 2010,14,17,18,19 |
| Tokyo Metropolitan Tama Medical Center | 2010,14,17,18,19 |
| Gifu Prefectural General Medical Center | 2012,14,17,18,19 |
| Ichinomiya Municipal Hospital | 2012,14,17,18,19 |
| Nippon Medical School Tama Nagayama Hospital | 2012,14,17,18,19 |
| University of Yamanashi Hospital | 2012,14,17,18,19 |
| Yamanashi Prefectural Central Hospital | 2012,14,17,18,19 |
| Gifu University Hospital | 2010,12,14,17 |
| Hachinohe City Hospital | 2010,12,14,17 |
| Japanese Red Cross Kitami Hospital | 2010,12,14,17 |
| Japanese Red Cross Nagano Hospital | 2010,12,14,17 |
| Osaka Prefectural Nakakawachi Emergency and Critical Care Center | 2010,12,14,19 |
| Tokai University Hachioji Hospital | 2010,12,14,19 |
| Akita University Hospital | 2010,12,17,18 |
| Aichi Medical University Hospital | 2010,12,17,19 |
| Japanese Red Cross Tokushima Hospital | 2010,12,18,19 |
| Juntendo University Urayasu Hospital | 2010,12,18,19 |
| Tokai University Hospital | 2010,12,18,19 |
| Yamagata Prefectural Central Hospital | 2010,12,18,19 |
| Saga University Hospital | 2010,14,17,18 |
| Tsukuba Medical Center Hospital | 2010,14,17,18 |
| Sapporo City General Hospital | 2010,14,18,19 |
| Hyogo Prefectural Nishinomiya Hospital | 2010,17,18,19 |
| National Hospital Organization Hokkaido Medical Center | 2010,17,18,19 |
| Osaka University Hospital | 2012,14,17,18 |
| National Hospital Organization Kumamoto Medical Center | 2012,14,17,19 |
| Tohoku University Hospital | 2012,14,17,19 |
| Handa City Hospital | 2012,14,18,19 |
| Japanese Red Cross Kyoto Daini Hospital | 2012,17,18,19 |
| St. Mary’s Hospital | 2012,17,18,19 |
| Aidu Chuo Hospital | 2014,17,18,19 |
| Iizuka Hospital | 2014,17,18,19 |
| Ina Central Hospital | 2014,17,18,19 |
| Iwata City Hospital | 2014,17,18,19 |
| Kyoto University Hospital | 2014,17,18,19 |
| National Center for Global Health and Medicine | 2014,17,18,19 |
| Nihonkai General Hospital | 2014,17,18,19 |
| Omihachiman Community Medical Center | 2014,17,18,19 |
| Ota Medical Hospital | 2014,17,18,19 |
| Saitama Medical University International Medical Center | 2014,17,18,19 |
| Shinshu University Hospital | 2014,17,18,19 |
| Sunagawa City Medical Center | 2014,17,18,19 |
| Tokyo Women's Medical University Medical Center East | 2014,17,18,19 |
| Toyama University Hospital | 2014,17,18,19 |
| Gifu Prefectural Tajimi Hospital | 2010,12,14 |
| Isesaki Municipal Hospital | 2010,12,14 |
| Iwate Medical University Hospital | 2010,12,14 |
| Keio University Hospital | 2010,12,14 |
| Matsudo City General Hospital | 2010,12,14 |
| Naha City Hospital | 2010,12,14 |
| Shizuoka Saiseikai General Hospital | 2010,12,14 |
| Tottori University Hospital | 2010,12,14 |
| National Defense Medical College Hospital | 2010,12,17 |
| St. Marianna University School of Medicine Hospital | 2010,12,18 |
| Chiba University Hospital | 2010,12,19 |
| Urasoe General Hospital | 2010,14,17 |
| Showa University Hospital | 2010,14,18 |
| Fujisawa City Hospital | 2010,14,19 |
| Jichi Medical University Saitama Medical Center | 2010,17,19 |
| Kurume University Hospital | 2010,18,19 |
| Hiroshima Prefectural Hospital | 2012,14,17 |
| National Hospital Organization Disaster Medical Center | 2012,14,17 |
| National Hospital Organization Mito Medical Center | 2012,14,17 |
| Tokuyama Central Hospital | 2012,14,17 |
| Japanese Red Cross Ise Hospital | 2012,14,19 |
| Toho University Omori Medical Center | 2012,14,19 |
| Fukuyama City Hospital | 2012,17,18 |
| Hyogo Emergency Medical Center | 2012,17,18 |
| Mie Prefectural General Medical Center | 2012,17,18 |
| Yokohama City University Medical Center | 2012,17,18 |
| Kyorin University Hospital | 2012,18,19 |
| Teine Keijinkai Hospital | 2012,18,19 |
| Showa University Fujigaoka Hospital | 2014,17,19 |
| Kansai Medical University Hospital | 2014,17,18 |
| Yokosuka General Hospital Uwamachi | 2014,17,18 |
| Sapporo Medical University Hospital | 2014,17,19 |
| Mie University Hospital | 2014,18,19 |
| The University of Tokyo Hospital | 2014,18,19 |
| Osaka Police Hospital | 2017,18,19 |
| Tosei General Hospital | 2017,18,19 |
| Juntendo University Nerima hospital | 2017,18,19 |
| Kitakyushu General Hospital | 2017,18,19 |
| Kushiro City General Hospital | 2017,18,19 |
| National Hospital Organization Takasaki General Medical Center | 2017,18,19 |
| Osaka City General Hospital | 2017,18,19 |
| Shonan Kamakura General Hospital | 2017,18,19 |
| Toyama Prefectural Central Hospital | 2017,18,19 |
| Yokkaichi Municipal Hospital | 2017,18,19 |
| Yokohama Minami Kyosai Hospital | 2017,18,19 |
| Numazu City Hospital | 2010,12 |
| Kanazawa University Hospital | 2010,12 |
| Hamamatsu University Hospital | 2010,12 |
| Japanese Red Cross Nagahama Hospital | 2010,12 |
| Japanese Red Cross Wakayama Medical Center | 2010,12 |
| Kawasaki Medical School Hospital | 2010,12 |
| Kitakyushu City Yahata Hospital | 2010,12 |
| Kobe University Hospital | 2010,12 |
| Nagasaki University Hospital | 2010,12 |
| Nagoya City University Hospital | 2010,12 |
| National Hospital Organization Kanmon Medical Center | 2010,12 |
| National Hospital Organization Kure Medical Center | 2010,12 |
| Seirei Mikatahara General Hospital | 2010,12 |
| Gunma University Hospital | 2010,14 |
| Kimitsu Chuo Hospital | 2010,14 |
| Osaka General Medical Center | 2010,14 |
| Shimane Prefectural Central Hospital | 2010,14 |
| Tsuyama Chuo Hospital | 2010,14 |
| Kawaguchi Municipal Medical Center | 2010,17 |
| Noto General Hospital | 2010,17 |
| Okinawa Prefectural Nanbu Medical Center & Children's Medical Center | 2010,17 |
| Kansai Medical University Medical Center | 2010,18 |
| Nippon Medical School Musashi Kosugi Hospital | 2010,18 |
| National Hospital Organization Osaka National Hospital | 2010,19 |
| Saiseikai Yokohamashi Tobu Hospital | 2010,19 |
| Chuno Kosei Hospital | 2012,14 |
| Japanese Red Cross Kumamoto Hospital | 2012,14 |
| Japanese Red Cross Takayama Hospital | 2012,14 |
| Kakogawa West City Hospital | 2012,14 |
| Saiseikai Shiga Hospital | 2012,14 |
| Seirei Hamamatsu General Hospital | 2012,14 |
| Toyota Memorial Hospital | 2012,14 |
| Wakayama Medical University Hospital | 2012,14 |
| Chukyo Hospital | 2012,17 |
| Dokkyo Medical University Nikko Medical Center | 2012,17 |
| National Hospital Organization Yokohama Medical Center | 2012,19 |
| Okazaki City Hospital | 2012,19 |
| Saku Central Hospital | 2012,19 |
| Yamagata University Hospital | 2012,19 |
| National Hospital Organization Minami Wakayama Medical Center | 2014,17 |
| Rinku General Medical Center | 2014,17 |
| Japanese Red Cross Kochi Hospital | 2014,18 |
| Fujita Health University Hospital | 2014,18 |
| Steel Memorial Hirohata Hospital | 2014,18 |
| Niigata Prefectural Shibata Hospital | 2014,19 |
| Kagawa University Hospital | 2017,18 |
| Okayama University Hospital | 2017,18 |
| South Miyagi Medical Center | 2017,18 |
| Taoka Hospital | 2017,18 |
| Sugita Genpaku Memorial Obama Municipal Hospital | 2017,19 |
| Eastern Chiba Medical Center | 2017,19 |
| Nagoya Ekisaikai Hospital | 2017,19 |
| Asahikawa Medical University Hospital | 2018,19 |
| Fujieda Municipal General Hospital | 2018,19 |
| Fukui Prefectural Hospital | 2018,19 |
| Funabashi Municipal Medical Center | 2018,19 |
| Japanese Red Cross Narita Hospital | 2018,19 |
| Kochi Health Sciences Center | 2018,19 |
| Oita University Hospital | 2018,19 |
| Okinawa Prefectural Chubu Hospital | 2018,19 |
| Saiseikai Utsunomiya Hospital | 2018,19 |
| Chiba Emergency Medical Center | 2010 |
| Japanese Red Cross Otsu Hospital | 2010 |
| Kanazawa Medical University Hospital | 2010 |
| Kindai University Hospital | 2010 |
| Kitasato University Hospital | 2010 |
| Niigata City General Hospital | 2010 |
| Niigata University Medical & Dental Hospital | 2010 |
| Nippon Medical School Chiba Hokusoh Hospital | 2010 |
| Osaka Medical College Hospital | 2010 |
| Osaki Citizen Hospital | 2010 |
| Saitama Medical Center | 2010 |
| University of Tsukuba Hospital | 2010 |
| Yokohama City University Hospital | 2010 |
| Fujisawa City Hospital | 2012 |
| Fukuoka University Hospital | 2012 |
| Dokkyo Medical University Hospital | 2012 |
| Nara Medical University Hospital | 2012 |
| National Hospital Organization Osaka National Hospital | 2012 |
| Saiseikai Kumamoto Hospital | 2012 |
| Saitama Medical University Hospital | 2012 |
| The Jikei University Hospital | 2012 |
| Toho University Ohashi Medical Center | 2012 |
| Tokyo Metropolitan Hiroo General Hospital | 2012 |
| Almeida Memorial Hospital | 2014 |
| Japanese Red Cross Nagoya Daiichi Hospital | 2014 |
| Kindai University Nara Hospital | 2014 |
| Showa General Hospital | 2014 |
| Hiroshima University Hospital | 2014 |
| Japanese Red Cross Kyoto Daiichi Hospital | 2014 |
| Japanese Red Cross Nagoya Daini Hospital | 2014 |
| Kanto Rosai Hospital | 2014 |
| Kishiwada Tokushukai Hospital | 2014 |
| Kouseiren Takaoka Hospital | 2014 |
| Kumamoto University Hospital | 2014 |
| Sendai City Hospital | 2014 |
| The Jikei University Daisan Hospital | 2014 |
| Yokosuka Kyosai Hospital | 2014 |
| Tane General Hospital | 2017 |
| Ibaraki Seinan Medical Center Hospital | 2017 |
| Iwate Prefectural Ofunato Hospital | 2017 |
| Japanese Red Cross Asahikawa Hospital | 2017 |
| Japanese Red Cross Fukaya Hospital | 2017 |
| Japanese Red Cross Nasu Hospital | 2017 |
| Kagoshima City Hospital | 2017 |
| Kariya Toyota General Hospital | 2017 |
| Kobe City Medical Center General Hospital | 2017 |
| Kyoto City Hospital | 2017 |
| Mishuku Hospital | 2017 |
| Miyazaki Prefectural Nobeoka Hospital | 2017 |
| Onomichi General Hospital | 2017 |
| Saiseikai Fukuoka General Hospital | 2017 |
| St. Marianna University School of Medicine Yokohama City Seibu Hospital | 2017 |
| Tokyo Medical and Dental University, Medical Hospital | 2017 |
| Uji Tokushukai Hospital | 2017 |
| University of the Ryukyus Hospital | 2017 |
| Uonuma Kikan Hospital | 2017 |
| Asahi General Hospital | 2018 |
| Iseikai Hospital | 2018 |
| Konan Kosei Hospital | 2018 |
| National Hospital Organization Kumamoto Medical Center | 2018 |
| National Hospital Organization Nagoya Medical Center | 2018 |
| Sapporo Higashi Tokushukai Hospital | 2018 |
| Tokushima University Hospital | 2018 |
| Tokyo Medical University Hospital | 2018 |
| Ehime Prefectural Central Hospital | 2019 |
| Ehime Prefectural Niihama Hospital | 2019 |
| Gifu University Hospital | 2019 |
| Hiroshima City Hiroshima Citizens Hospital | 2019 |
| Hyogo Prefectural Awaji Medical Center | 2019 |
| Japanese Red Cross Shizuoka Hospital | 2019 |
| Kasugai Municipal Hospital | 2019 |
| Kyushu University Hospital | 2019 |
| Nagoya University Hospital | 2019 |
| Odawara Municipal Hospital | 2019 |
| Tokushima Prefectural Miyoshi Hospital | 2019 |
| Tokyo Women's Medical University Hospital | 2019 |
| Tokyo Women's Medical University Yachiyo Medical Center | 2019 |
| Yokohama Rosai Hospital | 2019 |
